# B Chromosomes in Free-Living Flatworms of the Genus *Macrostomum* (Platyhelminthes, Macrostomorpha)

**DOI:** 10.3390/ijms222413617

**Published:** 2021-12-19

**Authors:** Kira S. Zadesenets, Nikolay B. Rubtsov

**Affiliations:** The Federal Research Center Institute of Cytology and Genetics SB RAS, 630090 Novosibirsk, Russia; rubt@bionet.nsc.ru

**Keywords:** B chromosome, karyotype variation, inheritance pattern, crossing experiment, interspecific hybridization, whole-genome duplication, flatworms

## Abstract

B chromosomes (Bs) or supernumerary chromosomes are extra chromosomes in the species karyotype that can vary in its copy number. Bs are widespread in eukaryotes. Usually, the Bs of specimens collected from natural populations are the object of the B chromosome studies. We applied another approach analyzing the Bs in animals maintained under the laboratory conditions as lines and cultures. In this study, three species of the *Macrostomum* genus that underwent a recent whole-genome duplication (WGD) were involved. In laboratory lines of *M. lignano* and *M. janickei*, the frequency of Bs was less than 1%, while in the laboratory culture of *M. mirumnovem*, it was nearer 30%. Their number in specimens of the culture varied from 1 to 14. Mosaicism on Bs was discovered in parts of these animals. We analyzed the distribution of Bs among the worms of the laboratory cultures during long-term cultivation, the transmission rates of Bs in the progeny obtained from crosses of worms with different numbers of Bs, and from self-fertilized isolated worms. The DNA content of the Bs in *M. mirumnovem* was analyzed with the chromosomal in situ suppression (CISS) hybridization of microdissected DNA probes derived from A chromosomes (As). Bs mainly consisted of repetitive DNA. The cytogenetic analysis also revealed the divergence and high variation in large metacentric chromosomes (LMs) containing numerous regions enriched for repeats. The possible mechanisms of the appearance and evolution of Bs and LMs in species of the *Macrostomum* genus were also discussed.

## 1. Introduction

B chromosomes (Bs) or supernumerary chromosomes are extra chromosomes in respect to the chromosomes from complement A (As, A chromosomes) and can vary in copy number from one to several dozen [1,2]. They are not essential for the normal development and fitness of their carrier as the chromosomes of basic karyotype. The Bs can be present in some individuals of a species in different copy numbers and be completely absent in others. Such variation was observed within a population or even within an individual [3,4]. The frequency, morphology, and DNA content of the Bs are usually species-specific, but Bs in various populations of the same species could be different, forming the unique systems of Bs [5,6,7]. There are species with tissue-specific B chromosome distribution. In goatgrass, *Aegilops speltoides*, Bs are absent in the roots and present in other plant parts [8]. Another example of the tissue-specific distribution of Bs is the germline-restricted chromosome (GRC) in songbirds, where GRC is eliminated from somatic cells during early embryogenesis [9,10]. Like Bs, GRC demonstrates high variability in size ranging from the dot-like chromosome in the karyotype in some species to the largest chromosome in others [10]. Both GRCs and other Bs are proposed to have originated from A chromosomes or their fragments [1,11]. The various mechanisms for accumulation of Bs were described, including nondisjunction and meiotic drive [12]. The phenomenon of B chromosome accumulation in cells of germline or in gametes is more widespread in animals. Probably, it allows avoiding the loss of Bs in gametes and in generations [13]. The post-meiotic drive (nondisjunction) is common in plants, while the preferential segregation of multivalent Bs occurs more often in animals [13]. In some plants and animals, the maintenance of B chromosome number is considered as a consequence of the opposite impact of the As and Bs on the B chromosome transmission to the next generation. For example, Bs show the positive drive in gametogenesis, while As suppress the increase in the number of Bs [13]. In maize, the accumulation of Bs was described as a result of the nondisjunction of B chromosome chromatids during the second pollen mitosis. Then, a cell containing two chromatids of the B chromosome preferably merges with the egg. Another cell that lost the B chromosome merges with the egg less often [14]. Three hypotheses for the B chromosomes’ maintenance are usually considered. (1) Bs have positive effects on the carrier’s phenotype when they occur in low numbers [15], and they negatively affect their carrier’s phenotype when they are present in high numbers. (2) The hypotheses of the parasitic nature of Bs—the Bs are selfish genetic elements with a harmful or neutral effect on host fitness [16]. (3) The Bs are beneficial for the host fitness and show a lack of drive [17]. Bs can be very different. We suppose that although most Bs are probably parasitic genetic elements, there are Bs that correspond to the other hypotheses mentioned above. There are numerous observations supporting all these hypotheses. Moreover, in many species, Bs often show non-Mendelian inheritance [12]. In experimental crosses, Bs are transmitted in higher frequencies than expected, leading to their offspring accumulation. Therefore, the irregular non-Mendelian inheritance could generate the variation of Bs in sizes, numbers, and even structural variation among the natural populations [11,18].

Usually, Bs mainly consist of repetitive DNA and are characterized as C-positive elements of karyotype. However, Bs containing C-negative regions with repeats distribution typical for euchromatic regions were also revealed [19,20]. C-positive Bs contain mainly repetitive DNA such as transposable elements (TEs), satellite DNA, ribosomal DNA, and organellar DNA [1,21]. However, DNA sequences derived from As, including protein-coding genes and pseudogenes were also discovered in the Bs of some species and the transcription of some protein-coding genes was shown [22,23,24].

The Bs are widespread. Among animals, they were found in nearly 800 species, including almost all the studied taxa [2]. However, up to now, among the large taxa only in birds, Bs were not found, though bird karyotypes included numerous microchromosomes that could mask small Bs impeding B chromosome identification. The number of species with Bs is obviously underestimated due to the many poorly studied taxonomic groups. Platyhelminthes including 23–40.000 species present one such poorly studied group. The reasons for the low number of karyotyped flatworm species are their features such as the small body sizes of most species, the complex life cycles in parasite worms, and the problem of their collection from the natural populations. The limited number of field-collected specimens prevented the correct estimation of B chromosome distribution among flatworms. Nevertheless, Bs were found in both free-living and parasitic flatworms [25,26,27,28,29]. In the studied species, the numbers of Bs revealed per sample varied from 0 to 10 [25]. For example, in most karyotyped species of trematodes (*Echinostoma revolutum*, *Apatemon gracilis*), 1–2 copies of Bs per the sample were revealed, while in other species (*Diplodiscus subclavatus* and *Notocotylus* sp.), up to 10 copies of Bs per sample were found [25]. Interestingly, no Bs were found in monogenean ectoparasites and cestods [25]. In general, the number of known species with the Bs in the taxon correlates with the number of karyotyped species and the number of studied natural populations of these species. In many species, descriptions of the Bs were performed only with routine cytogenetic techniques such as Giemsa- or DAPI staining of metaphase spreads.

The alternative approach in B chromosome studies includes the analysis of B chromosome carrying specimens cultivated as a laboratory stock or an inbred line. It appeared to be effective in studies of Bs in plants, for instance in rye *Secale cereale* L, maize *Zea mays*, and goatgrass *Aegilops speltoides* [8,30,31]. We used a similar approach in the present study of the Bs in three *Macrostomum* species. The analysis of Bs in the laboratory stock cannot be used for the description of B chromosome distribution in natural populations. However, these laboratory lines can be applied as laboratory models for studies of prerequisites of B chromosome emergence, their origin, possible direction, and mechanisms of B chromosome evolution, positive or negative selection of the individuals with different Bs, and maintenance of the various numbers of Bs it carries.

Considering that polyploidy is widespread among the flatworms in which Bs were described, in this study, we included the neopolyploid species analyzing their laboratory lines and cultures characterized with karyotype instability. In the genus *Macrostomum*, three species, namely, *M. lignano*, *M. janickei*, and *M. mirumnovem*, make up a very intriguing group [32,33,34]. Namely, all of them underwent a recent whole-genome duplication (WGD) [33,35]. Moreover, according to a phylogenetic tree of the genus *Macrostomum*, two independent events of genome duplication have taken place. One of them took place in the phylogenetic lineage of *M. lignano*/*M. janickei*, while another happened in the phylogenetic lineage of *M. mirumnovem* [33,36]. After genome doubling, a similar karyotype reorganization took place in their evolution. The chromosomes of the ancient set fused into one large metacentric chromosome [32,33]. In the phylogenetic lineage of *M. lignano*/*M. janickei*, the inversions were revealed in paralogous chromosome regions [35]. During the speciation of *M. janickei*, the number of large chromosomes doubled [37]. Previously, we suggested that genomes of *M. lignano* and *M. janickei* have resulted from autopolyploidization, while in the *M. mirumnovem* phylogenetic lineage, the interspecific hybridization followed by genome doubling took place [33]. Interspecific hybridization facilitated a burst of transposable elements (TE). TE activation, including the intense expansion and amplification of TEs, provided prominent genome instability [38,39]. In the phylogenetic lineage of *M. mirumnovem*, the modern genome arose due to intensive DNA amplification in the large chromosome derived from the fusion of ancient chromosomes that led to the emergence of regions enriched for DNA repeats. Besides the development of regions enriched for DNA repeats, karyotype instability included numerical chromosomal abnormality and structural chromosomal rearrangements such as deletions and inversions in large chromosomes. Additionally, numerous Bs were present in the studied worms [33,40]. Based on the results of the earlier studies of Bs performed in other species, we suggested that Bs in *M. mirumnovem* reflect the genome and karyotype destabilization taking place after large-scale genome reorganization, such as a WGD and massive chromosome fusions. Today, the questions regarding B chromosome origin, DNA content, and their influence on the carrier’s phenotype became actual. In this study, we assessed the distribution of Bs in the laboratory lines and cultures of *M. lignano*, *M. janickei*, and *M. mirumnovem.* We analyzed the distribution of the Bs among the worms of the laboratory lines during long-term cultivation, the transmission rates of Bs in the progeny obtained from crosses of worms with different numbers of Bs, and from self-fertilized isolated worms. We estimated the possible impact of the B chromosome number on worm fertility, attempted to analyze the correlation between the numbers of Bs and large As. The latter was also conducted for the progeny of self-fertilized individuals with known karyotypes.

## 2. Results

### 2.1. Identification of Bs in the Laboratory of M. lignano, M. janickei and M. mirumnovem

**Figure 1:**
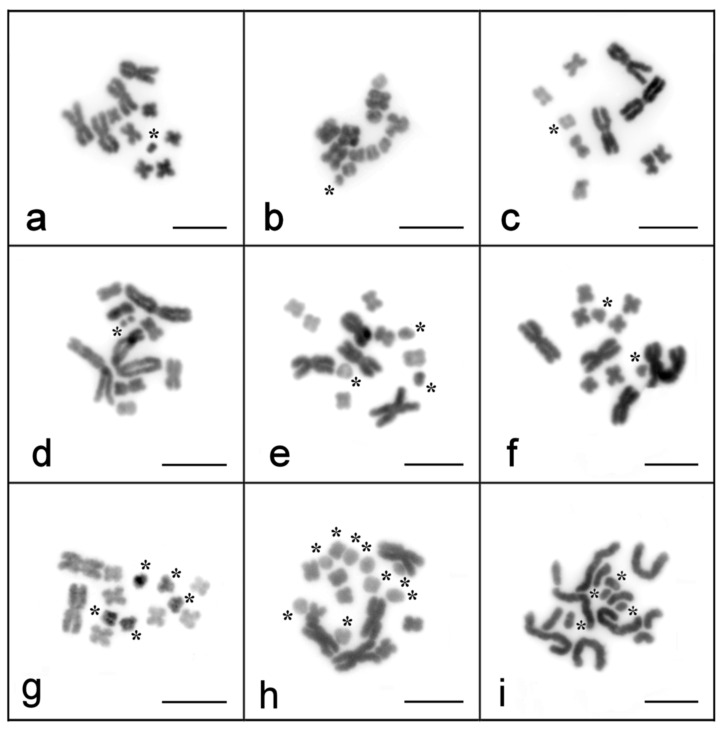
Metaphase spreads of *M. lignano* (**a**), *M. janickei* (**b**), *M. mirumnovem* (**c**–**i**). The metaphase spreads were obtained in 2017 (**c**,**d**), 2018 (**e**,**f**), and 2020 (**a**,**b**,**g**–**i**). B chromosomes are marked with an asterisk. Scale bar 10 µm.

In the laboratory lines of *M. lignano* and *M. janickei*, we found few specimens with B chromosomes. Among the karyotyped worms of *M. lignano* (312 individuals), we detected the only worm with a small extra chromosome (Figure 1a; Table 1). Two worms, both carrying one small extra chromosome, were revealed among 200 specimens of *M. janickei* (Figure 1b; Table 1). The revealed B chromosomes were present in all the analyzed metaphase spreads obtained from the specimens. We obtained no results suggesting mosaicism on the Bs in these species. The found Bs were tiny, and the description of their morphology was impossible.

Karyotyping of the laboratory culture of *M. mirumnovem* appeared to be more complex and surprising. After three months of cultivating field-collected worms in the laboratory, we karyotyped for the first time the specimens of *M. mirumnovem* [33]. At this stage, we discovered high karyotype diversity among the cultivated worms. We detected seven individuals having tiny extra chromosomes, referred to as Bs [33]. Besides these Bs, the numbers of other chromosomes also varied. In most of the 52 karyotyped worms (48 specimen; 92.3%), we observed three copies of large metacentrics and varying numbers of small chromosomes. Among the other worms, we found one with one large metacentric (1.9%), two specimens with four (3.8%), and one specimen with six (1.9%) large metacentrics. According to Chromosomal In Situ Suppresion (CISS) hybridization using Whole Chromosome Paints (WCPs), the small metacentrics included three pairs of small metacentric As and various numbers of Bs similar to the small As in size [33].

**Figure 2:**
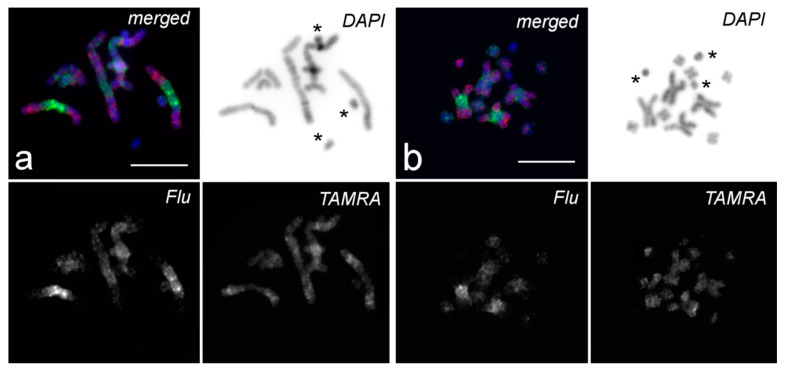
Painting patterns of metaphase chromosomes of *M. mirumnovem* after CISS-hybridization with PCP *Mmi2med* (Flu, green) and PCP *Mmi2dist* (TAMRA, red). DNA staining with DAPI (blue). (**a**) Bs are not painted with the DNA probes; (**b**) one B carries specific fluorescent signal from PCP *Mmi2dist.* Bs are marked with an asterisk. Scale bar 10 µm.

In contrast to the typical painting patterns of small As, the painting with WCPs derived from large metacentrics gave Bs either a very bright signal or no signal at all. The WCPs derived from small As did not paint Bs [33]. Painting with Partial Chromosome Paints (PCPs), generated from the proximal region and the distal regions of one of the large metacentrcis (further called LMs) (the MMI2 chromosome) PCP *Mmi2med* and PCP *Mmi2dist*, respectively [33], was carried out for Bs characterization (Figure 2). The results of the performed CISS-hybridization suggested that Bs were enriched for chromosome-specific repeats. In the case of these DNA repeats present in the PCPs, painting was provided on the Bs’ very intensive signal, while the absence of such repeats in the PCPs led to the complete absence of a FISH signal. Furthermore, in the last case, we did not observe the background signal that is typical of some C-negative regions of As. Therefore, considering the particular painting patterns of Bs using the PCPs, we supposed that the Bs consisted mainly of repetitive DNA specific to large metacentrics.

All the Bs identified by the CISS-hybridization of WCPs were small chromosomes showing different sizes and morphologies. Their sizes varied from very small—dot-like—to nearly the size of small As. According to the centromere position, some of them were metacentrics, and others were submetacentrics or even subacrocentrics. Unfortunately, we could not determine the centromere position in dot-like Bs. The analysis of the morphology of Bs identified using the painting provided by the CISS-hybridization of PCPs and WCPs in 32 specimens allowed us to determine the characteristic features of Bs for their identification in routine karyotyping with DAPI staining. However, we cannot exclude the possibility that identifying some Bs could be difficult. This concerns the Bs that were near the small As in size.

**Figure 3:**
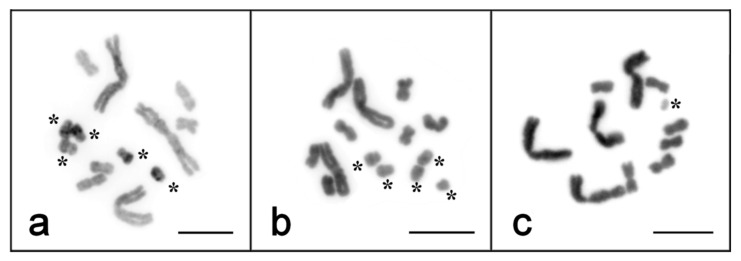
Heterogeneous morphology and sizes of B chromosomes among the specimens of *M. mirumnovem*. B chromosomes are marked with an asterisk. Scale bar 10 µm. (**a**) four of five Bs have intensively painted AT-rich regions in the q-arm; (**b**) the detected five Bs have different sizes; (**c**) one tiny B chromosome is much smaller than the smallest A chromosome.

Nevertheless, they were usually a little smaller than the small As or were submetacentrics (Figure 3). Some of these Bs showed more intensive DAPI-staining than the small As, suggesting that they were enriched with AT-rich DNA, or their chromatin was more condensed in mitosis (Figure 3a).

### 2.2. As and Bs in the Laboratory Culture of M. mirumnovem during Long-Term Cultivation

Based on the single-worm karyotyping data, we distinguished three chromosome subsets in the *M. mirumnovem* karyotype. Three pairs of small metacentric As present one ‘stable’ subset. Most of the karyotyped worms (97.96%) contained six small metacentrics in size and morphology corresponding to standard small As. Among the 244 karyotyped worms, only five specimens showed a lower number of small As. Three of them had five small As instead of six and two individuals appeared to be haploids (two large and three small As). These haploid worms showed normal morphological and behavioral treats. Unfortunately, we did not study their fertility.

Two other chromosome subsets were variable. One of them consisted of large metacentrics (LMs) and their derivatives. Earlier, we categorized most of them into two types, namely, the MMI1 and MMI2 chromosomes, which differed a little in size from each other [33]. Their derivatives that resulted from structural rearrangements were large chromosomes of different sizes and morphologies. Among the karyotyped specimens, the number of LMs varied from one to nine. The specimen with one LM was found in 2017. The number and variations of the LMs increased over time during their cultivation in the laboratory (Figure 1c–i).

The third chromosome subset that consisted of Bs was described in the previous section. The Bs showed a high level of variation in size, morphology, and DNA content. The average number of Bs per specimen grew substantially over the years of their cultivation (Table 2).

In 2020, no worm without a B chromosome was found. Furthermore, we detected a variable number of Bs in cells of the same specimen. Mosaicism on Bs was revealed in 52.17% of the karyotyped worms. In 21 specimens, we also revealed mosaicism on LMs (Table 2). Two of them showed a stable number of Bs (six and eight). Other 19 specimens had mosaics on both the As and Bs. All the worms that had mosaics on the LMs contained a high number of Bs in their karyotype, four Bs or more per specimen. We used the highest number of Bs detected in the mosaic specimen for its description, supposing that the loss of the B chromosome in worm development was a more plausible event that its gain. However, the gain of an additional B chromosome cannot be excluded. We have to note that identifying small As and some Bs with morphology similar to the small As could be difficult and less reliable in mosaic worms. As a result, some of the mosaics on small As could remain unrecognized.

We want to emphasize that all the karyotyped worms have shown a normal phenotype, suggesting that the variation of B chromosomes and the large metacentric number have no obvious effect on the worm phenotype.

### 2.3. Inheritance Patterns of Large As and Bs in M. mirumnovem

Nine of the ten pairs of *M. mirumnovem* worms produced a total of 338 hatchlings; the number of the F1 offspring varied from 9 to 48 per cross over a period of three weeks (Table 3). We detected mortality in the F1 offspring (hatchlings or juveniles that died before maturation) (Table 3). One pair of *M. mirumnovem* worms (cross #10) gave no progeny. All of the parental worms except specimen #10.2 showed a standard set of small As (three pairs of small metacentrics). The karyotype of specimen #10.2 consisted of four LMs, nine identical small metacentrics, and a variable number of Bs (7–10). It presumably contained a triple set of small As. Another worm in this cross, specimen #10.1, had a standard karyotype, four LMs, three pairs of small metacentrics, and six Bs. We supposed that a triple set of small As could lead to fertility disturbance. The hatchlings from other crosses were allowed to mature, then they were karyotyped (Table 3).

In the F1 offspring obtained in the crossing experiment, we revealed nearly the same level of mosaicism on the Bs (49.14%) as was determined with the last karyotyping of the worms from the laboratory culture of *M. mirumnovem* (52.2%). Due to the small body size of the *M. mirumnovem* worms, we could analyze a limited number of metaphase spreads per specimen, which was, in general, 10–15. Thus, the karyotyping allowed mosaicism of a high level, but not the peculiarities of B chromosome mosaicism, to be detected.

Besides the mosaicism on Bs, we discovered mosaicism on LMs. It was revealed in the laboratory culture (22.83%) and the F1 offspring produced by crosses (23.27%). The number of LMs usually varied from three to five chromosomes in both the parents and offspring. In a few cases, we found only two large non-homologous chromosomes, probably the MMI1 and MMI2, which differed slightly in their size and DNA content. We suppose that the presence of at least one copy of the MMI1 and one copy of the MMI2 was essential for the normal development and growth of the *M. mirumnovem* worms [30]. However, it should not be forgotten that one specimen with one LM in its karyotype was found in 2017.

Further analysis was based on the following suggestions: (1) karyotypes of all specimens should contain at least one copy of the MMI1 and MMI2; (2) pairs of homologous chromosomes are conjugated and normally segregated into gametes; (3) an unpaired chromosome is transmitted into gametes randomly. To predict the possible number of LMs in the gametes of worms from crosses, the identification of chromosomes MMI1 and MMI2 was not required. Worms with two LMs should contain one MMI1 and one MMI2. They did not conjugate and were transmitted into gametes randomly, providing gametes with different numbers of LMs (from zero to two). Worms with three LMs should contain one pair of homologous LMs and one unpaired LM. Homologous LMs should conjugate and segregate normally, while an unpaired LM should transmit into gametes randomly, providing gametes with one or two LMs. The karyotype with four LMs could contain four homologous LMs or one pair of homologous LMs and two unpaired LMs. In the gametes of such specimens, we should expect a varied number of LMs (from one to three). The maximal number of LMs in the specimens involved in the crossing experiments was five. In these specimens, the LMs could include two pairs of homologous LMs and one unpaired. The minimal expected number of LMs in the gametes of these specimens could be two, while the maximal number could be three. We calculated the expected numbers of LMs for the F1 offspring from each cross (Table 3). The number of LMs in the F1 siblings of all the crossings except crossing #2 corresponded to the expected numbers of LMs. Cross #2 was the exception. The expected maximal number of LMs in F1 siblings of this crossing was four, while in karyotypes of the studied specimens, it is appeared to be higher (up to six).

As was shown earlier, *M. mirumnovem* is an outcrossing and self-fertilizing species [41]. We analyzed the transmission of Bs and LMs to the next generation by karyotyping the progeny produced by self-fertilized worms. Self-fertilization was registered in 17 out of the 56 isolated worms (30.36%) [41]. In this study, we karyotyped the F1 offspring of four self-fertilized worms. One of them was the only self-fertilized specimen out of the 24 isolated specimens of *M. mirumnovem* (Table S1). Three other worms were isolated from the F1 offspring produced by crosses (one worm from cross #3 and two others from cross #12). The number of offspring from self-fertilized worms varied from three to ten (Table 4). The main results of their karyotyping are shown in Table 4. The detailed descriptions of karyotypes are presented in Table S2.

To estimate the expected B and LM numbers, we used the same approach as was used for the analysis of B and LM transmission in crossings of karyotyped animals. Considering the self-fertilization in the parental worms, we have considered karyotyped offspring as progeny from two parents with identical karyotypes. In one of the self-fertilizing worms, the numbers of Bs were different from the expected. The karyotype of the parent was mosaic on Bs and LMs. The number of Bs varied from one to two, while LMs varied from three to four. The expected number of Bs in offspring was 0–4, but in one specimen of its offspring, Bs varied from seven to nine. The other offspring obtained from this self-fertilized worm showed an expected number of Bs (from one to three). We suppose that these data indicate the possibility of B chromosome accumulation in cells of the germline.

## 3. Discussion

### 3.1. Peculiarities of Genome and Karyotype Organization in the Laboratory Lines of M. lignano, M. janickei and M. mirumnovem

In their evolutionary history, the genomes and karyotypes of all the species involved in the study underwent a recent WGD followed by chromosome rearrangements provided by one large metacentric chromosome containing all the chromosomes of an ancient haploid set. However, the mechanisms of genome doubling in the *M. lignano*/*M. janickei* and *M. mirumnovem* lineages were probably different. We suppose that in the *M. lignano*/*M. janickei* lineage, the ancestral genome was doubled via autopolyploidization, while in the *M. mirumnovem* lineage, interspecific hybridization occurred [33].

The last suggestion derived from a high frequency of structural chromosome rearrangements, with the appearance of new chromosome regions enriched for DNA repeats, and de novo Bs observed in *M. mirumnovem* karyotypes. In both the *M. lignano* and *M. janickei* karyotypes, variation was associated mainly with the copy numbers of the large chromosome [32,33]. In the *M. mirumnovem* karyotype, chromosome reorganization and copy number variation were associated mainly with large metacentrics and Bs. The chromosome rearrangements and expansion of DNA repeats were mainly restricted by large metacentrics. Three pairs of small A chromosomes remained relatively stable in this species. This is similar to the subgenomes in allopolyploid plants when TE activation and chromosome rearrangements occur, preferably in one of the subgenomes [42,43].

The generally accepted mechanism of B chromosome formation includes (1) a small extra chromosome appearing containing the pericentromeric region of the original A chromosome (proto-B); (2) following insertions of DNA fragments in proto-B, accompanied by their amplification. Another suggested mechanism includes the formation of regions enriched for DNA repeats in the original A chromosome followed by deletions of the euchromatic regions located between such regions [7,20]. The analysis of chromosome organization in *M. mirumnovem* also revealed numerous structural rearrangements in large metacentrics and the amplification of DNA repeats in their regions [33]. The reorganization of LMs could be induced with TE activation, which is the consequence of interspecific hybridization and the conflict between diverged parental genomes [44]. This suggestion is in good accordance with the described expansion of TEs and their amplification, which is limited to one of the subgenomes in some studied allopolyploids [45]. LMs in *M. mirumnovem* could represent a similar subgenome derived from one of the ancient parental genomes and was involved in massive genome reshuffling. Their intense reorganization included DNA amplification, regions appearing enriched for repetitive DNA (possibly TE-associated regions) and the loss of some regions between them. Such reorganization could provide a great diversity of large As in the modern *M. mirumnovem* genome. It could also induce the appearance of numerous new Bs.

### 3.2. The Bs in M. mirumnovem

Unfortunately, none of the specimens collected directly from natural populations were available for karyotyping. We firstly performed the karyotyping of the *M. mirumnovem* worms in the three months after establishing its laboratory culture. We revealed Bs of different sizes and morphologies in 30% of the studied specimens. It is possible that, in *M. mirumnovem*, the frequency of Bs and their variability differ in various natural populations [2]. This study analyzed the possible scenarios for B chromosome evolution, behavior, and association with a basic genome of *M. mirumnovem* on the example of Bs in a long-term cultivated laboratory culture.

The variation of the Bs in size from dot-like to small metacentrics of a basic karyotype allowed us to make some suggestions. It is probable that Bs originated from the pericentromeric regions of As such as human small supernumerary marker chromosomes (sSMC) and the Bs of many other species [46]. However, in contrast to human sSMC, they were increased in size by DNA amplification. This is a common mechanism for the B chromosome evolution. The recent WGD in the evolutionary history of *M. mirumnovem* probably included interspecific hybridization, inducing TE activity and, therefore, chromosome instability accompanied by local DNA amplification. All of these events facilitated the appearance of prerequisites for B chromosome formation and their further evolution. In some species, Bs could exceed the largest A chromosome of karyotype in their size. However, in *M. mirumnovem*, an unknown mechanism prevented the size of Bs increasing. Instead of the size increase, we observed the increasing B chromosome number per specimen and the frequency of worms with Bs. The DNA fragments amplified in Bs were probably also amplified in LMs, forming chromosome regions enriched for repeats [33]. We suppose that there is a mechanism that prevents the formation of very large regions consisting of DNA fragments intensively amplified in the *M. mirumnovem* genome. From this point of view, sequencing DNA from Bs became an actual task.

After a long-term cultivation of the *M. mirumnovem* culture, we detected an increase in the number of B chromosomes per specimen and an increased frequency (up to 100%) of B-carrying worms. We tried to analyze B chromosome transmission to the next generation. From the results of the crossing experiment, we obtained no evidence for B chromosome accumulation in cells of germline or gametes. The distribution of Bs in gametes looked random, but more analyzed crossings are required for a conclusion. The data obtained from the self-fertilized animals showed that an accumulation of Bs is possible. However, this was revealed in only one of the four self-fertilized worms. Namely, we found offspring from the self-fertilized mosaic worm with 1–2 Bs whose karyotype contained an unexpectedly high number of B chromosomes (7–9 Bs). These data indicated possible B chromosome accumulation in the cells of the germline. Even the transmission of both chromatids of both Bs into a gamete and the fusion of such gametes would lead to eight Bs. The appearance of the ninth B chromosome should require additional processes such as the accumulation of Bs in germline cells or errors in mitosis after fertilization. Further studies are required to analyze the meiotic drive and accumulation of Bs in the germline cells or even their precursors.

An additional opportunity for B chromosome accumulation might be associated with the very high regenerative potential of *M. mirumnovem*. After the amputation of a large part of its body, the worm can recover its whole body, including testes and ovaries. The revealed mosaicism on Bs raises questions about the possible changing of the chromosome set in the cells taking part in gametogenesis after regeneration. However, even if this mechanism exists, it cannot play a significant role in B chromosome evolution. Additionally, we would like to highlight the fact that the first mosaic worm was found in the *M. mirumnovem* culture only after long-term cultivation in the laboratory.

Positive and negative selection could be the important factor influencing the number of B chromosomes in worms. In the absence of a strong meiotic drive, positive selection could lead to an increase in B chromosome numbers in cultivated *M. mirumnovem* worms. However, the variation of Bs in size, morphology, and DNA content significantly complicates the estimation of selective pressure on the number of B chromosomes. Based on the obtained data, it is possible to suggest that the selective pressure cannot be very high.

### 3.3. The LMs in M. mirumnovem

Besides Bs, we observed another set of variable elements in the *M. mirumnovem* karyotype. In 2017, the karyotypes of most analyzed specimens contained three LMs. However, over time, their number and copy number variation increased. Earlier, LMs were referred to as the chromosomes MMI1 and MMI2, and differed slightly in size, morphology and DNA content. Derivatives of these chromosomes were also observed [33].

Nevertheless, we identified at least one copy of MMI1 and one of MMI2 in almost all the studied worms. The only exception was found in 2017. LMs consisted of alternated euchromatic regions and regions enriched for repeats. They are obligatory elements of the *M. mirumnovem* karyotype and, for this reason, were referred to As.

On another side, their number varied in the studied specimens and their average number per specimen increased after long-term cultivation in the laboratory. Earlier, we described the copy number variation of the large chromosome in the inbred line of *M. lignano* [32]. We studied the transmission of extra copy(ies) of the large chromosome to the progeny from crosses between worms with 2–4 copies. We observed no significant deviation from the expected results in the F1 offspring. Similar experiments performed on the *M. mirumnovem* worms revealed unexpected copy numbers of LMs in the F1 from one of the nine crosses. In karyotypes of both parents, three large metacentrics were identified. Based on cytogenetic data, we supposed that they were MMI1 and MMI2. We suggested that homologous chromosomes should be conjugated and normally segregated in gametes, while the remaining chromosome can be randomly transmitted. Thus, the number of large metacentrics in the gametes of these parental worms could be one or two; therefore, in the F1 offspring, the number of LMs should vary from two to four. Unexpectedly, the actual number of LMs in the offspring varied from two to six. We distinguished between MMI1 and MMI2 according to the difference in their size. However, we cannot exclude the possibility that LMs of a similar size could differ in their structure and DNA content. In this case, they might distribute into gametes independently. Considering that two different LMs are essential for normal worm development, the expected numbers of LMs could vary from two to six. Based on the obtained data, we suppose that more different LMs could exist in the laboratory culture. These data prompted us to revise the suggestions listed above. An increased number of LMs could result from a disturbance in meiosis derived from the impairment of ‘homologous’ chromosome conjugation and the random transmission of all the LMs.

The differences between copies of LMs could lead to meiotic disturbance and increasing chromosome variation in gametes. The great variability of observed LM sets in analyzed specimens was probably formed by several processes occurring in the culture of *M. mirumnovem*. One of them could be the meiotic drive mentioned above and the preferential transmission of large chromosomes into oocytes.

For an explanation of the obtained results, we make the following suggestions:

1. Three pairs of small metacentrics contained the basic conservative subgenome of the *M. mirumnovem* genome;
2. Some essential genes are also located in LMs;
3. After WGD, LMs derived from a chromosome formed by fusing the chromosomes of one of the parental species;
4. Due to the loss of different regions from the original large chromosome, its derivatives contained different sets of genes;
5. Only several different LMs (such as MMI1 and MMI2) contained a complete set of genes required for the normal development of worms;
6. Progressive degradation of LMs and their divergence required an increased number of different LMs for all of the essential genes to be present in the genome.

Thus, selective pressure favoring requiring combinations of different LMs could become a significant force of karyotype evolution. The suggestions listed above allowed us to explain the finding of the specimen with one LM in its karyotype. It was probably a less degraded LM containing all the essential genes.

We have to note that increasing the differences between large metacentrics could facilitate their random transmission to the next generation and the formation of new variants through further chromosome rearrangements. At the same time, selection in favor of animals with a complete set of genes could increase the number of LMs and retain their less degraded copies.

We should note that features of LMs such as copy number variation and random transmission to the next generation are similar to those observed for Bs. Furthermore, the evolution of LMs included the formation of regions enriched for repeats and the loss of the regions located between them [33]. This is in good accordance with one of the suggested mechanisms of B chromosome formation [20]. However, LMs or their sets remain essential for the existence of *M. mirumnovem*, and they are obligatory chromosome elements for the normal development of the *M. mirumnovem* worms. They look like the intermediate stage between As and Bs.

### 3.4. Mosaicism on Large Metacentrics and Bs

The first mosaic worms on LMs and Bs were revealed after the long-term cultivation of the *M. mirumnovem* culture. Unexpectedly, in 2020, the frequency of mosaic worms appeared to be rather high and involved, besides Bs, the large metacentric chromosomes. We suppose that during the long-term cultivation of *M. mirumnovem*, new type(s) of Bs arose that increased the frequency of disturbance in mitosis. In humans, a high frequency of mosaicism occurs before embryo implantation [47,48]. Later, mosaic embryos or their aneuploid cells could be eliminated. At what stage and in what way mosaics were forming in *M. mirumnovem* should be elucidated. In *M. mirumnovem*, they can affect the karyotype of the next generation. The chromosomal mosaicism in *M. mirumnovem* restricted with Bs and LMs consisted of or enriched for repetitive DNA. They could be heterochromatic and probably showed features of heterochromatin as late DNA replication. The very large size of heterochromatin could probably increase the frequency of mitotic disturbance and the loss of chromosomes containing large heterochromatic regions.

### 3.5. Futured Perspectives on Studies of Bs in M. mirumnovem

The long-term cultivation of *M. mirumnovem* worms could also facilitate karyotype instability and the numerous and structural variability of Bs and LMs. Prerequisites for such intensive karyotype changing should originate from the natural population from which the specimens were collected. From this point of view, the laboratory culture of *M. mirumnovem* worms is a very convenient and efficient model for studying the origin and regularities of the evolution of Bs. The perspective directions of B chromosome studies include cytogenetic analysis of meiotic chromosomes. This could discover the particularities of B chromosome behavior in meiosis and elucidate the mechanism of B chromosome transmission to the next generation. Unfortunately, we faced difficulties in meiotic chromosome preparation in the species that underwent recent WGD, *M. lignano*, *M. janickei*, and *M. mirumnovem*. Chromosome preparation in other species from the *Macrostomum* genus provided numerous spreads of pachytene chromosomes, while in the pos-WGD *Macrostomum* species, only a few pachytenes were obtained. We suppose that some unusual features in the meiosis of these species prevent us from obtaining spreads of the meiotic cells. We are working on developing a protocol to obtain spreads of pachytene chromosomes in these species.

Other approaches in the study of Bs include the generation of microdissected DNA libraries and DNA probes from a single copy of Bs followed by the sequencing of the DNA library and FISH with a generated DNA probe [49,50]. Analysis of the data obtained with sequencing of the microdissected DNA library generated from the B chromosome required genome assemblies of *M. mirumnovem* or closely related species. Considering the astonishing genome and karyotype instability in *M. mirumnovem*, it is challenging to assemble a good-quality genome draft, especially in the absence of the sequenced genome of its diploid, a closely related species. Studies on the generation of microdissected DNA probes for the FISH experiments and the generation of DNA libraries for their subsequent sequencing are in progress.

## 4. Materials and Methods

### 4.1. Study Organisms

*M. lignano* Ladurner, Schärer, Salvenmoser and Rieger 2005, and *M. janickei* Schärer 2019, and *M. mirumnovem* Schärer and Brand 2019 are free-living hermaphroditic flatworms of the genus *Macrostomum*. *M. lignano* and *M. janickei* are closely related species [36]. Both species are characterized by a karyotype polymorphism, mainly associated with the copy number of the large chromosome [32]. The specimens of *M. lignano* used for the current study were taken from the laboratory inbred line DV1 that is commonly used for different studies [51,52,53,54,55]. The specimens of *M. janickei* were taken from the outbred laboratory culture [32,37]. Both *M. lignano* and *M. janickei* are out-crossing, reciprocally fertilized species [56,57].

*M. mirumnovem* is an out-crossing species that is able to self-fertilize [41]. The specimens of *M. mirumnovem* were taken from an outbred culture established in 2017 [36]. Earlier, we showed the unusual karyotype organization in *M. mirumnovem* associated with its genome instability [33].

The laboratory lines and cultures of *M. lignano*, *M. janickei* and *M. mirumnovem* were maintained in the laboratory at 20°C in Petri dishes containing f/2 medium (32‰) and fed with the diatom algae *Nitzschia curvilineata* [36,58,59].

### 4.2. Inheritance Pattern of Bs in M. mirumnovem

The same-aged hatchlings of *M. mirumnovem* were obtained, as was described earlier [59] and transferred according to the design experiment. To assume the frequency of self-fertilization, 24 hatchlings of *M. mirumnovem* were isolated in a 24-well-plate (one hatchling per well with 1,0 mL of 32‰ ASW and diatom algae); in parallel, 24 hatchlings (crosses #1–12) were paired randomly in another 24-well-plate. After their maturation, the well-plates with the paired and single worms were checked for the progeny every three days (for three weeks). The hatchlings found were transferred separately into new well-plates (one hatchling per well) and kept until maturation. Then, the karyotypes of both parents and at least 10 worms from their progeny were checked (except for cross#8, where only nine siblings were produced). We did not check the karyotypes of the parental worms before the crossing experiment because we could obtain contradictory results due to the suggested somatic instability in *M. mirumnovem*. Additionally, we described the karyotypes of isolated single worms. In addition, we karyotyped all worms in progeny from self-fertilizing worms. To determine the chromosome number of individual worms, we used a single-worm karyotyping technique.

### 4.3. Single-Worm Karyotyping Technique

Chromosome slide preparation was performed using a single worm karyotyping technique [32]. Karyotype diversity in the laboratory-reared *Macrostomum* species (*M. lignano*, *M. janickei*, and *M. mirumnovem)* was previously described [32,33]. Additionally, we karyotyped 92 specimens from the laboratory culture of *M. mirumnovem*. This was performed to enable us to assume the karyotype stability of *M. mirumnovem* and its tendency to change.

### 4.4. Fluorescence In Situ Hybridization

Microdissected DNA probes (Partial Chromosome Paints, PCPs Mmi2med and Mmi2dist) were obtained earlier [33]. The obtained DNA probes were labeled Flu-dUTP or TAMRA-dUTP (BioSan, Novosibirsk) in additional PCR cycles. FISH experiments were performed with the DNA probes on mitotic metaphase spreads of *M. mirumnovem* according to the standard protocol [33].

### 4.5. Chromosome Staining and Microscopy Analysis

Chromosome slides were counterstained with the fluorescent dye DAPI, 4’,6-diamidino-2-phenylindole, dissolved in the antifade solution (VectaShield, Vector Laboratories, Burlingame, CA, USA). Stained metaphase chromosomes and results of fluorescent in situ hybridization (FISH) were captured using a CCD-camera installed on an Axioplan 2 compound microscope (ZEISS Oberkochen, Germany) equipped with filter cubes #49, #10, and #15 (ZEISS, Oberkochen, Germany) using AxioVision (Zeiss, Oberkochen, Germany) or ISIS4 (METASystems GmbH, Altlussheim, Germany) software at the Center for Microscopic Analysis of Biological Objects of SB RAS (Novosibirsk, Russia) (#0259-2021-0011).

## Figures and Tables

**Table 1:** Number of B carriers among the worms of the laboratory-reared *Macrostomum* species.

Species/ Year	B Carrying Worms, N (%)		References
	0	≥1B	
*M. lignano* (DV1)			
2014	134	0	[32]
2015	78	0	[32]
2020	100	1 (1%)	This study
*M. janickei*			
2018	100	0	[33]
2020	100	2 (2%)	[33]
*M. mirumnovem*			
2017	52	7 (dot-like Bs; 13.46%) 8 (B of enlarged size 15.38%)	[33]
2018	100	37 (37%)	[33]
2020	92 *	92 (100%)	This study

* Additionally, 158 specimens were karyotyped in crossing experiment (paired and isolated worms and siblings obtained in crosses and from self-fertilized individuals).

**Table 2:** The karyotyped worms with different number of Bs and LMs in the laboratory culture of *M. mirumnovem*.

Year	N *	N, with Bs	N of Mosaics, Bs	N of Mosaics, LMs **	N of Mosaics, Bs, LMs	Range of Bs	N of Worms with 1B	N of Worms with Bs	Mean Number of Bs per Worm
2017	52	15 (28.85%)	0	0	0	1–2	9 (17.3%)	6 (11.54%)	0.48 ± 0.244
2018	100	37 (37%)	0	0	0	1–10	2 (2%)	35 (35%)	1.55 ± 0.475
2020	92	92 (100%)	48 (52.17%)	21 (22.83%)	19 (20.65%)	1–11	1 (1.09%)	91 (98.9%)	5.90 ± 0.433

* Total number of randomly selected and karyotyped specimens per year; ** large metacentrics.

**Table 3:** A chromosome set and B chromosomes in the F1 offspring produced by nine crosses between B-carrying worms of *M. mirumnovem*.

Thepair ID	The Worm ID	Karyotype	A Chromosomes		Bs	Offspring (Total/Gone), N	Karyotyped Worms, N	Range of Numbers of Large As per Worm		Mean Number of Large As	Range of Numbers of Bs	Mean Number of Bs per Worm
			Large, N	Small, N				Expected	Observed			
1	1.1	2n = 9 + 3Bs	3	6	3	48	10	2–4	2–4	3.1 ± 0.46	1–8	4.8 ± 1.39
	1.2	2n = 8–10 + 8Bs	2–4	6	8	-	-	-	-	-	-	-
2	2.1	2n = 9 + 4Bs	3	6	4	40	10	2–4	2–6	3.6 ± 0.84	1–7	4.5 ± 1.06
	2.2	2n = 9 + 4–5Bs	3	6	4–5	-	-	-	-	-	-	-
3	3.1	2n = 9 + 4Bs	3	6	4	46	10	3–4	2–5	4 ± 0.29	1–7	3.8 ± 1.12
	3.2	2n = 10 + 4Bs	4	6	4	-	-	-	-	-	-	-
4	4.1	2n = 8–9 + 6–7Bs	2–3	6	6–7	44	10	2–3	2–4	2.6 ± 0.43	4–9	6.7 ± 0.66
	4.2	2n = 8 + 5–6Bs	2	6	5–6	-	-	-	-	-	-	-
5	5.1	2n = 9–10 + 2Bs	3–4	6	2	39/1	10	3–4	4–5	4.4 ± 0.32	3–7	5.3 ± 0.78
	5.2	2n = 10 + 7–8Bs	4	6	7–8	-	-	-	-	-	-	-
6	6.1	2n = 8 + 6Bs	2	6	6	40/3	10	2	2–4	2.5 ± 0.44	2–7	5.3 ± 0.88
	6.2	2n = 8 + 6Bs	2	6	6	-	-	-	-	-	-	-
7	7.1	2n = 10 + 7Bs	4	6	7	44/1	10	4–5	3–5	4.3 ± 0.42	3–8	5.6 ± 1.06
	7.2	2n = 11 + 2Bs	5	6	2	-	-	-	-	-	-	-
8	8.1	2n = 9–10 + 5–6Bs	3–4	6	5–6	9	7	3–4	3–4	3.86 ± 0.28	5–7	5.86 ± 0.51
	8.2	2n = 10 + 7Bs	4	6	7	-	-	-	-	-	-	-
10	10.1	2n = 10 + 6Bs	4	6	6	0	0	4	-	-	-	-
	10.2	2n = 13 + 7–10Bs?	4	9 *	7–10	-	-	-	-	-	-	-
12	12.1	2n = 10–11 + 6–7Bs	4–5	6	6–7	28/3	11	4–5	3–6	4.27 ± 0.59	1–6	3.73 ± 0.88
	12.2	2n = 10 + 1B	4	6	1	-	-	-	-	-	-	-

* Nine small chromosomes had similar sizes and morphologies.

**Table 4:** Karyotype diversity among the offspring from self-fertilized individuals.

ID Worm	Karyotype	As		Bs	Offspring (Total/Gone), N	Karyotyped Worms, N	Range of Numbers of Large As	Mean Number of Large As	Range of Numbers of Bs	Mean Number of Bs
		Large	Small							
S10	2n = 9–10 + 1–2Bs	3–4	6	1–2	10/2	8	3–5	4.13 ± 0.25	0–9	2.75 ± 1.81
3.8 *	2n = 9–10 + 0–1B	3–4	6	0–1	4/1	3	3–4	4	1–2	1.67 ± 0.65
12.15 *	2n = 11 + 3–4Bs	5	6	3–4	8	8	3–5	4.125 ± 0.25	1–4	2.75 ± 0.72
12.21 *	2n = 9–10 + 4–6Bs	3–4	6	4–6	9	8	3–6	4.13 ± 0.69	3–6	4.5 ± 0.74

* Specimens from isolated progeny F1 obtained from crosses.

## Supplementary Materials

The following are available online at https://www.mdpi.com/article/10.3390/ijms222413617/s1.

## Abbreviations

WGD whole-genome duplication; CISS chromosomal in situ suppression; LM large metacentric; GRC germline-restricted chromosomes; WCP whole chromosome paints; PCP partia chromosome paint; TE transposable element; sSMC small supernumerary marker chromosome.
