# Supplementary material for: B Chromosomes in Free-Living Flatworms of the Genus Macrostomum (Platyhelminthes, Macrostomorpha)
Source: Int J Mol Sci. 2021 Dec 19;22(24):13617. doi: 10.3390/ijms222413617 (PMC8708343; doi:10.3390/ijms222413617)
Supplement: Supplementary file 1 [file ijms-22-13617-s001.zip › Table S1.pdf]

Table S1: Karyotypes of isolated replicates of *M. mirumnovem*

ID	Karyotype	Large As	Small As	No. of Bs
S1	2n=9+6-7Bs	3	6	6-7
S2	2n=9+6Bs	3	6	6
S3	2n=8-9+4-6Bs	2-3	6	4-6
S4	2n=10+6-7Bs	4	6	6-7
S5	2n=10+5Bs	4	6	5
S6	2n=8+7Bs	2	6	7
S7	2n=9+3Bs	3	6	3
S8	2n=9+7Bs	3	6	7
S9	2n=10+6-7Bs	4	6	6-7
S10*	2n=9-10+1-2Bs	3-4	6	1-2
S11	2n=8+5-7Bs	2	6	5-7
S12	2n=9-10+4Bs	3-4	6	4
S13	2n=10+6Bs	4	6	6
S14	2n=9-10+2Bs	3-4	6	2
S15	2n=10+7BS	4	6	7
S16	-	-	-	-
S17	2n=10+9Bs	4	6	9
S18	2n=8-10+4-6Bs	2-4	6	4-6
S19	2n=10+3-4Bs	4	6	3-4
S20	2n=10+3Bs	4	6	3
S21	2n=8-10+8-9Bs	2-4	6	8-9
S22	2n=9+4Bs	3	6	4
S23	2n=9-10+7Bs	3-4	6	7
S24	2n=9-10+6-8Bs	3-4	6	6-8

*self-fertilized worm (see Table S2)
