# Supplementary material for: B Chromosomes in Free-Living Flatworms of the Genus Macrostomum (Platyhelminthes, Macrostomorpha)
Source: Int J Mol Sci. 2021 Dec 19;22(24):13617. doi: 10.3390/ijms222413617 (PMC8708343; doi:10.3390/ijms222413617)
Supplement: Supplementary file 1 [file ijms-22-13617-s001.zip › Table S2.pdf]

Table S2: List of karyotype variants revealed among the F1 offspring from the self-fertilized worms of *M. mirumnovem*.

ID worm	Karyotype	A chromosomes		Bs	Comments
		large	small		
S10	2n=9-10+1-2Bs	3-4	6	1-2	
S10.1	-	-	-	-	gone
S10.2	2n=9-10+1-2Bs	3-4	6	1-2	
S10.3	2n=10-11+7-9Bs	4-5	6	7-9	
S10.4	2n=10+1-2Bs	4	6	1-2	
S10.5	2n=10+0-1B	4	6	0-1	
S10.6	2n=10+2Bs	4	6	2	
S10.7	2n=10+3Bs	4	6	3	
S10.8	-	-	-	-	died
S10.9	2n=10+0-1B	4	6	0-1	
S10.10	2n=10+0-2Bs	4	6	0-2	
3.8	2n=9-10+0-1B	3-4	6	0-1	
3.8.1	2n=10+1B	4	6	1	
3.8.2	2n=10+2Bs	4	6	2	
3.8.3	2n=9-10+1-2Bs	3-4	6	1-2	
12.15	2n=11+3-4Bs	5	6	3-4	
12.15.1	2n=10+3-4Bs	4	6	3-4	
12.15.2	2n=11+4Bs	5	6	4	
12.15.3	2n=10+3Bs	4	6	3	
12.15.4	2n=10+2Bs	4	6	2	
12.15.5	2n=10+1B	4	6	1	
12.15.6	2n=10+2Bs	4	6	2	
12.15.7	2n=10+2-3Bs	4	6	2-3	
12.15.8	2n=9-10+2-3Bs	3-4	6	2-3	
12.21	2n=10-11+3-5BS	4-5	6	3-5	
12.21.1	2n=9-10+4-6Bs	3-4	6	4-6	
12.21.2	2n=8-10+5Bs	3-4	5-6	5	
12.21.3	-	-	-	-	died
12.21.4	2n=10+5Bs	4	6	5	
12.21.5	2n=11+3Bs	6	5	3	
12.21.6	2n=9+3Bs	3	6	3	
12.21.7	2n=9+4Bs	3	6	4	
12.21.8	2n=9-10+3-5Bs	3-4	6	3-5	
12.21.9	2n=10-11+4-5Bs	4-5	6	4-5	
